# Musical auditory feedback BCI: clinical pilot study of the Encephalophone

**DOI:** 10.3389/fnhum.2025.1592640

**Published:** 2025-06-16

**Authors:** Thomas A. Deuel, James Wenlock, Alana McGovern, James Rosenthal, Juan Pampin

**Affiliations:** ^1^Swedish Neuroscience Institute, Seattle, WA, United States; ^2^Department of Digital Art and Experimental Media (DXARTS), University of Washington, Seattle, WA, United States; ^3^Department of Statistics, University of Washington, Seattle, WA, United States; ^4^Department of Electrical Engineering, University of Washington, Seattle, WA, United States

**Keywords:** brain computer interface, music feedback system, motor disability, motor rehabilitation, motor imagery

## Abstract

**Introduction:**

Therapeutic strategies for patients with severe acquired motor disability are relatively limited and show variable efficacy. Innovative technologies such as brain-computer interfaces (BCIs) have been developed recently that might benefit certain types of patients.

**Methods:**

Here, we tested a previously described auditory BCI, the Encephalophone, which may offer new options to improve quality of life and function. Eleven subjects with acquired moderate to severe motor disability, who had lost their ability to express themselves musically, were enrolled and 10 completed a clinical pilot study of the hands-free Encephalophone brain-computer interface (BCI). Subjects were briefly instructed on the use of the Encephalophone BCI, which uses EEG measured motor imagery to allow users to generate musical notes in real time without requiring movement. Subjects then underwent a pitch-matching task, a measure of accuracy, to attempt to match a given target pitch 3 times within 10 s. They were allowed free play, where they could improvise music over a backing track. After 2–3 songs - approximately 10 min - of freely improvised playing, subjects repeated the pitch-matching task. There were 3 sessions of testing and free play per subject, within 2 weeks, with at least 1 day separating sessions.

**Results:**

All subjects, on average, improved their pitch-matching accuracy by 15.6 percentage points and increased their number of hits by 58.7% over the 3 sessions, with all subjects scoring accuracy percentages significantly above random probability (19.05%). A subjective self-reporting survey of ratings of such factors as a feeling of expressing oneself, enjoyment, discomfort, and feeling of control showed a generally favorable response.

**Discussion:**

We suggest that this training approach using an auditory BCI may provide an innovative solution to challenges in recovery from motor disability.

**Clinical trial registration:**

https://research.providence.org/clinical-research, Swedish Health Services #: STUDY2017000301.

## Introduction

Brain-Computer Interface (BCI) research has been ongoing for more than 50 years now. The vast majority of BCI research has focused on visual feedback, rather than auditory feedback. Auditory, and specifically musical auditory feedback offers several tangible and potential advantages: (1) auditory feedback can be used in patients with visual impairment who might not be able to operate a visual feedback-based BCI (Choi et al., 2023), (2) auditory feedback may facilitate a stronger learning effect (McCreadie et al., 2013; Nijboer et al., 2008), and (3) auditory (and particularly musical) feedback may be more motivating for more sustained attention (Nijboer et al., 2010). Part of the relative lack of auditory feedback BCIs comes from an assumption that visual feedback is more robust than auditory feedback. Nonetheless, results of a limited number of studies comparing auditory vs. visual feedback demonstrate initially more accuracy with visual feedback, but with improved learning effect from auditory feedback and comparable rates of accuracy after several sessions (McCreadie et al., 2013; Nijboer et al., 2008). Approaches using auditory feedback have been insufficiently explored. Auditory feedback can be presented in a wide variety of manners, such as pure sine-wave tones, ‘natural’ sounds such as animal noises, pink noise (in which the power spectral density is inversely proportional to the frequency of the signal), or musical instruments. Auditory feedback P300 BCIs have compared ‘artificial’ sounds (e.g., beeps) to ‘natural’ sounds and found better BCI performance with ‘natural’ sounds (Choi et al., 2023). In the study of McCreadie and others, pink noise, anechoic cello, or anechoic percussion instruments were used for auditory feedback; participants preferred the instrument sounds to the pink noise (McCreadie et al., 2013).

Motivational factors have a significant effect on BCI performance (Nijboer et al., 2010), and music has a positive effect on task attention for simple tasks (Kiss and Karina, 2021). We therefore hypothesized that auditory feedback in BCI - specifically musical auditory feedback - may be more motivational and therefore more effective feedback for BCI performance of individuals than visual or non-musical auditory feedback.

We have previously shown that a motor imagery BCI using musical auditory feedback (the Encephalophone) could be operated by non-motor impaired healthy novices with accuracy significantly better than random (Deuel et al., 2017). Here, we sought to determine whether hospital patients with moderate to severe motor impairment in a clinical setting could also operate the Encephalophone with accuracy significantly better than random, and whether there was a learning effect with improvement of accuracy after 3 sessions. We additionally recorded subjective self-reporting of ratings of such factors as a feeling of expressing oneself, enjoyment, discomfort, and feeling of control.

## Methods

In this trial, 11 subjects with acquired moderate to severe motor disability in at least one upper extremity were enrolled, and 10 subjects completed, 3 sessions of testing and training on the Encephalophone auditory-feedback based BCI.

Patients were enrolled under informed consent after Institutional Review Board (IRB) review (ethics approval was by Swedish Health Services IRB number: STUDY2017000301) and approval under the following criteria: (a) acquired moderate to severe motor impairment (3/5 strength or worse on neurological exam), including but not limited to an upper limb, (b) sufficient cognition to be able to understand instructions on use of the Encephalophone, and (c) prior musical expression and performance ability which was impaired or eliminated due to acquired motor impairment. Prior musical expression and performance ability could have been professional or amateur, instrumental (e.g., piano, guitar, etc.) or vocal.

**Figure 1:**
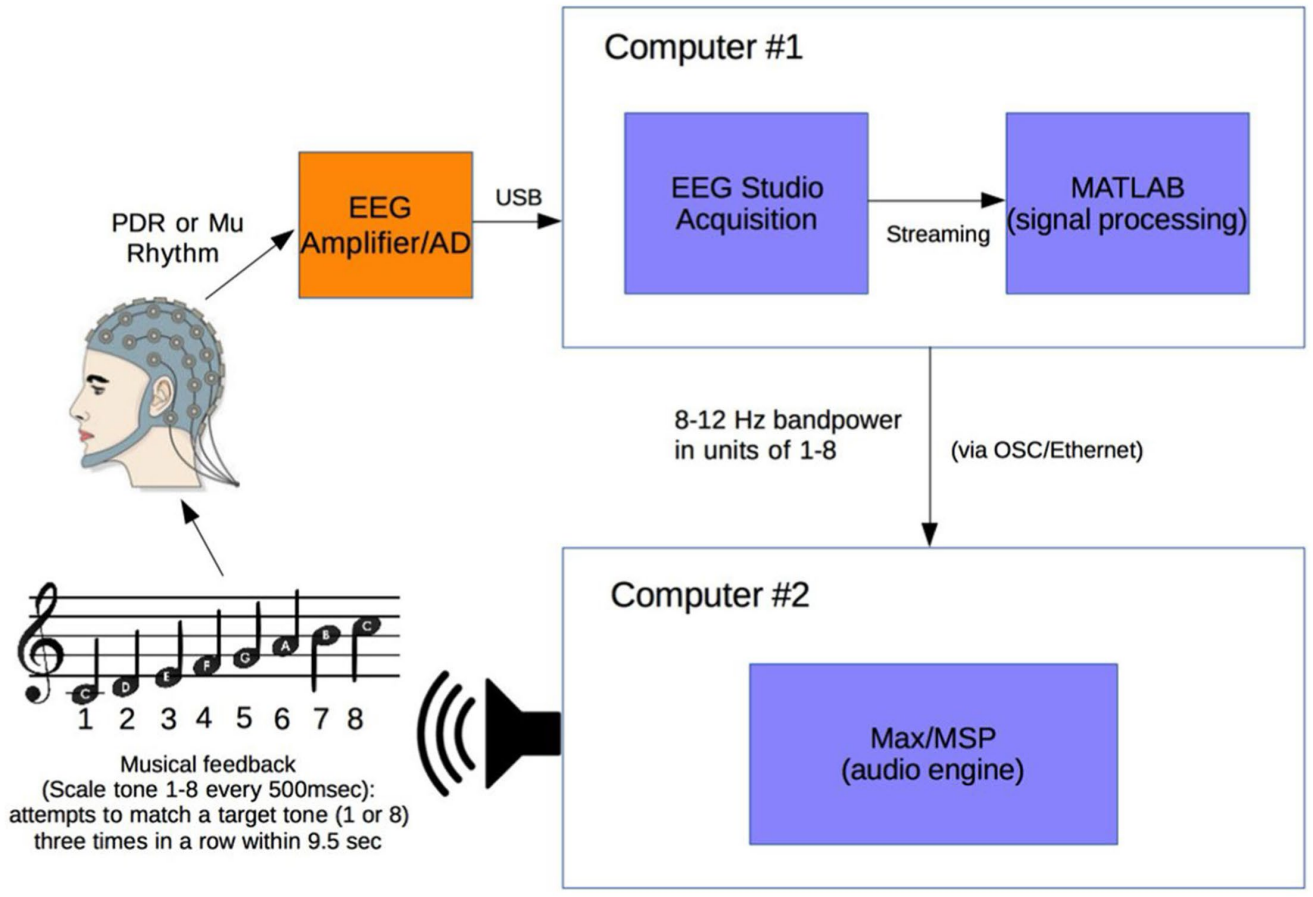
Experimental setup. Electroencephalogram (EEG) signal from subject wearing electrode cap is sent from Mitsar 201 EEG amplifier to Computer #1 where 8–12 Hz posterior dominant rhythm (PDR) or Mu power is converted to a value from 1 to 8. This value from 1 to 8 is sent via OSC to Computer #2 where it is converted to a musical piano tone in the key of C (seven tones of C major scale and octave, from C4 to C5). Subjects generating tones attempt to match them with a presented target tone [from Deuel et al. (2017)].

The design and function of the Encephalophone BCI has been previously described (Deuel et al., 2017; Figure 1), and EEG signal collection processing methods were identical in this study. A Mitsar 201 EEG (Mitsar Co., Ltd., St. Petersburg, Russia; distributed by Nova Tech, Inc. Mesa AZ USA) and 19-channel ElectroCap electrode cap (Electro-Cap International Inc., Eaton, OH USA) were used to collect EEG signal utilizing the International 10–20 system of electrode placement (American Electroencephalographic Society, 1994) from the 10 subjects.

Subjects were positioned in a relaxed, reclining position with a headrest to minimize muscle artifacts, and were positioned facing away from computer screens and other equipment to eliminate any potential for visual feedback. EEG signal at a sampling rate of 500 Hz was initially processed in a HP Pavilion PC (Hewlett-Packard, Palo Alto, CA) with Mitsar EEG Acquisition software, where filters were applied (100 Hz low-pass, 0.5 Hz high-pass, and 60 Hz notch filters). Raw EEG signal was visually verified by a physician clinical neurophysiologist for good signal quality and lack of artifacts. EEG data was then streamed in real time to Matlab (The MathWorks, Inc., Natick, MA) via the Mitsar Matlab API.

Matlab scripts for real-time signal processing were created to apply a fourth order Butterworth filter in the 8–12 Hz frequency band to generate an estimate of signal power for the sensorimotor cortex mu rhythm from bipolar electrodes F3-C3 (international 10–20 system) for right hand motor imagery. The delay in the system from EEG signal acquisition to Matlab processing was approximately 20 msec. The filter was applied to incoming segments of 500 msec of data. The bandpass filtered data was rectified and then averaged over the entire segment length to produce a single power estimate for every segment. Thus while not strictly in real-time, subjects were producing tones from the prior 500 msec of data with an approximate delay of 20 msec.

A calibration was created for each individual subject and each individual trial session of the Encephalophone. The 5 min long calibration period consisted of twenty 15 s long alternating cued states (“on” or “off”). For sensorimotor cortex mu rhythm, an auditory cue of “on” cued the awake, resting state, and “off” cued the motor imagery (but not actual movement) state: subjects were instructed to imagine right hand grasping and opening at a rate of approximately 1 Hz as per sensorimotor imagery BCI methods of Neuper et al. (2006). This calibration period established the range of values of 8–12 Hz signal power for an individual subject and individual trial session in the different cued states, then divided these values into eight equal sized ‘bins’, or ranges of values, based on the calibration period signal power histogram. After calibration, these 8 possible values generate the 8 scale degrees of the C major musical scale including the octave (C4 to C5).

After the calibration period is used to calibrate the instrument to each individual, the device enters the free-running period, during which a value from 1 to 8 is generated every 500 msec from the sensorimotor cortex 8–12 Hz frequency power of the user. Subjects were allowed brief (3 min) free-running practice with note generation before accuracy experiments.

This free-running stream of values from 1 to 8 in Matlab is sent at a rate of one value per 500 msec (120 bpm musical tempo for quarter notes) using OSC (Open Sound Control) along an Ethernet cable via a router to a second computer - an Apple MacBook Pro (Apple, Inc. USA) - where it is received by Max/MSP music generation software (Cycling ‘74, USA). The streaming values from 1 to 8 are used to generate the 8 scale degree notes in the C major musical scale with a synthesized piano tone: 8 notes from C4 (261.6 Hz) to C5 (523.3 Hz).

Each subject participated in 3 sessions with the Encephalophone, with at least 1 day between sessions, and no more than 2 weeks total to complete the 3 sessions. At the beginning of each session, the subject completed a 5 min note accuracy test using the Encephalophone. During this test, the subject was randomly presented with either a high or low C note (C5 or C4 on the piano). There are 8 possible notes the Encephalophone can produce and only the highest (note 8) and lowest (note 1) notes were presented during the test as target notes. The subject then attempted, using the Encephalophone, to emit the note that they hear (the target note) by thinking about different levels of movement. Every 0.5 s, the subject produced a note via the Encephalophone. The subject’s goal was to achieve the correct note or the adjacent note 3 times in a row within 9.5 s. If the subject succeeded, this was classified as a ‘hit’. For example, if a subject was presented with note 1, the subject needed to play either a 1 or a 2 three times in a row. When the subject succeeded or the 9.5 s elapsed, whichever happened first, a new target note (either the high or low note) was presented. This process repeated for 5 min. The primary dataset recorded for each test included the number of hits and the number of trials for each 5 min test. A subjects accuracy score was calculated as the percentage of ‘hits’ for each presented target note (hits divided by trials for each test). After this first 5-min test, the subjects had 20 min to use the Encephalophone freely and improvise over a backing track of music. Then they repeated the same 5-min test. With this study design, each of the 10 participants had 2 accuracy scores from each of the 3 sessions, totaling 6 accuracy scores for each participant. We refer to the tests taken at the beginning of each session as ‘pre-tests’ (Tests 1, 3 and 5) and those taken at the end as ‘post-tests’ (Tests 2, 4 and 6).

After calibration, the Encephalophone entered a ‘free play’ mode where a subject in the relaxed state would produce higher pitch notes, and the subject in motor imagery state would produce lower pitch notes in real time. There are 8 possible notes (a C major scale from C4 to C5) which could be generated by the subject.

When calibration was completed, each subject underwent an initial pitch matching test for accuracy in generating notes with the Encephalophone. As described above, in this 5 min long test, subjects are given one of two randomly chosen target notes (C4 or C5 note), then had 9.5 s to match the note (or its nearest neighbor) three times in a row. If they matched the note successfully (scored a hit) before 9.5 s, or were unsuccessful after 9.5 s (scored a miss), they were given another target note. Subjects tried to match as many target notes as possible within 5 min time.

After the first accuracy test (‘pre-test’), subjects played freely accompanied by pre-recorded backing tracks of music in various styles for 20 min. Subjects played music freely using the Encephalophone to attempt to improvise melodies over music in any pitches they desire. This was intended to be pleasurable and allow subjects to express themselves musically, but also intended to aid with learning to control the Encephalophone more effectively.

Subjects then underwent a second pitch matching test at the end of each session (‘post-test’), identical to the first.

At the end of the third session, subjects were asked to answer a questionnaire consisting of 5 questions on subjective reporting of their enjoyment, their feeling of being able to express themselves, as well as satisfaction, physical and emotional discomfort. These responses were rated from 1 (disagree strongly) to 5 (agree strongly).

## Results

Eleven subjects with acquired moderate to severe motor disability were enrolled in the study (Table 1). They varied in age from 18 years old to 85 years old, and had various etiologies for their motor disability, such as ischemic stroke, amyotrophic lateral sclerosis, multiple sclerosis, and spinal cord injury. One subject did not complete the trials and dropped out due to medical complications (this subject acquired a pneumonia, unrelated to this study, requiring cancelation of trial sessions); thus 10 subjects completed this clinical trial pilot study of the Encephalophone audio-based BCI.

**Table 1:** Trial subject demographics.

Subject	Age	Motor disability	Years Music	Instrument
1	50	ALS quad	4	Piano, drums, voice
2	27	Pontine stroke	4	Guitar
3	65	C4 traumatic cord	50	Piano
4	47	MS quad	1	Guitar, piano, trumpet
5	72	Right MCA stroke	0	Listening only
6	50	Right BG ICH	10	Drums, lesser guitar
7	40	ALS	6	Vocals, lesser clarinet
8	36	TBI	9	Flute
9	85	Left BG stroke	6	Piano, accordion
10	18	Transverse myelitis	12	Flute, singing
11*	78	Pontine stroke	2	Coronet

Age, etiology of motor disability, years of musical training, and instrument(s) of 11 trial subjects; *Subject 11 dropped out of the study due to medical complications unrelated to the study.

All 10 subjects improved accuracy percentage (defined as number of hits/number of trials per 5 min test) by the last session (Table 2).

**Table 2:** Individual accuracy percentages, with number of trials in parentheses.

Session	1		2		3	
Subject	Pre	Post	Pre	Post	Pre	Post
1	65% (51)	61% (44)	78% (58)	49%(43)	70% (47)	93.0% (57)
2	65% (46)	78% (45)	57% (47)	59%(44)	62% (50)	80% (56)
3	33% (39)	41% (42)	51% (45)	62%(47)	78% (55)	87% (70)
4	57% (49)	58% (43)	62% (55)	51%(41)	51% (43)	64% (55)
5	30% (37)	45% (40)	34% (38)	32%(38)	80% (54)	58% (50)
6	60% (48)	48% (42)	59% (44)	30%(37)	40% (40)	63% (48)
7	29% (38)	44% (41)	58% (48)	57%(44)	51% (53)	64% (63)
8	33% (45)	45% (40)	43% (42)	71%(56)	49% (43)	67% (45)
9	55% (47)	46% (41)	77% (53)	71%(56)	52% (48)	58% (50)
10	52% (46)	55% (47)	55% (53)	65%(52)	71% (48)	77% (57)
Average	48%	52%	58%	55%	60%	71%

**Figure 2:**
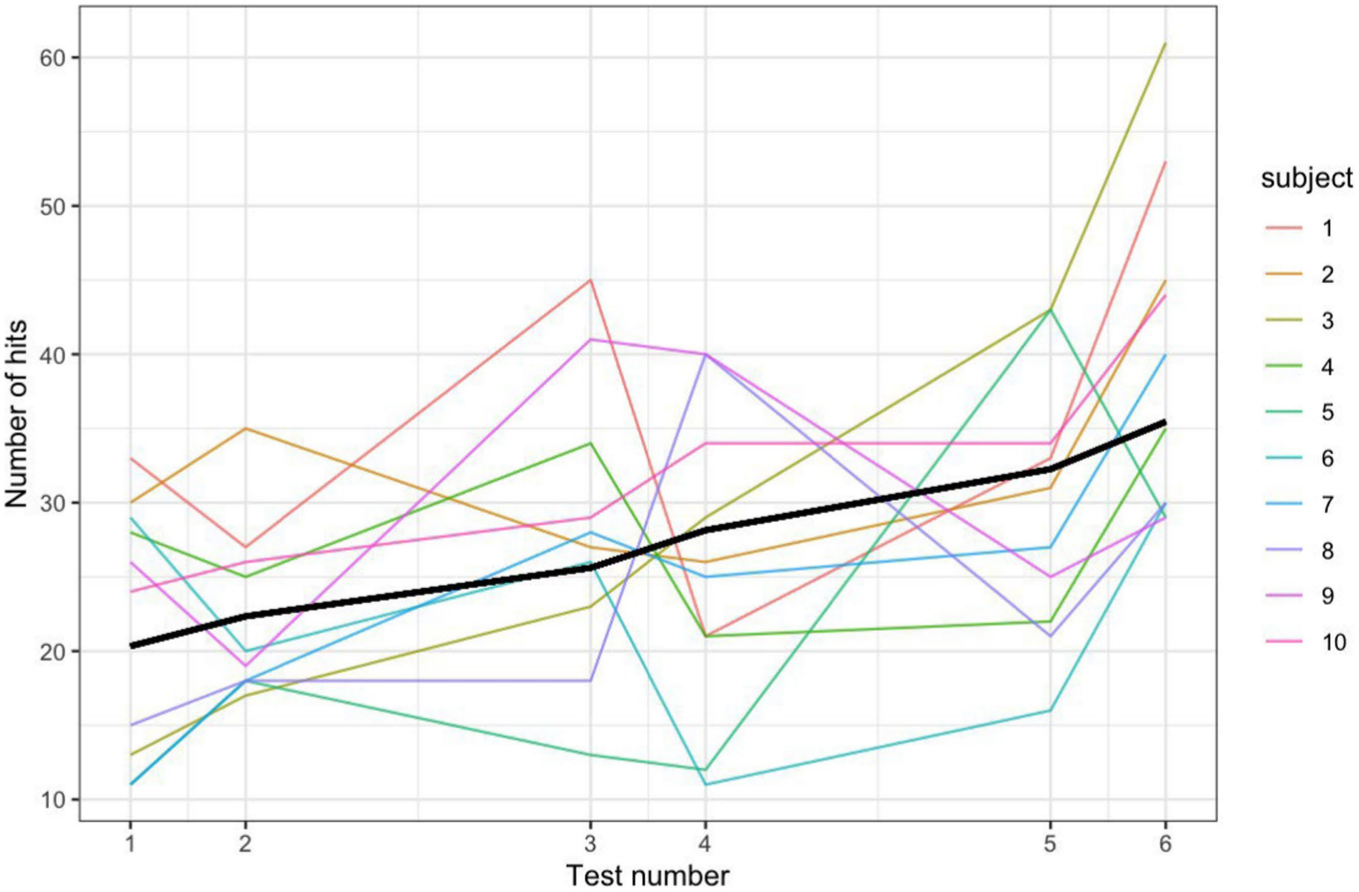
Note matching number hits for 3 sessions with a Poisson GLMM fit line.

Because of the structure of the test, the more quickly a participant gets a hit, the more trials they will get and their total number of hits may be higher. Therefore, ‘number of hits’ may capture aspects of performance not captured by accuracy percentage. For both outcome measures (accuracy percentage and number of hits) we used mixed effects regression models. In each model, we include a set of fixed effects for the session number and another set for whether the test was a pre- or post-test. We also include subject-level random intercepts and slopes to account for subject-level variability. Since the average number of hits over time is a count variable, it was modeled using a generalized linear mixed effects model (GLMM, Figure 2), in which the outcome is assumed to follow a Poisson distribution. The 10 subjects increased their number of hits on average by 58.7% over the 3 sessions.

**Figure 3:**
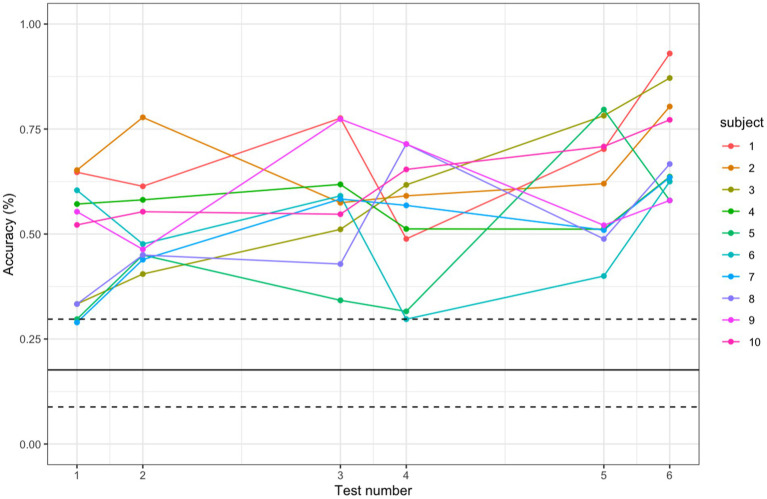
Comparing participant accuracy to accuracy of random note generator. Accuracy scores of each participant for each test, with the bold black line representing the median score of the random note generator and the dashed lines representing the 5th and 95th percentiles of the random note generator score.

The average accuracy percentage over time was modeled using a linear mixed model (LLM) in which the outcome is assumed to follow a normal distribution. Note that we do not use a Binomial distribution because the number of trials is not independent from accuracy (i.e., higher scores will have a higher number of trials). The 10 subjects increased their accuracy percentage (hits/trials per 5 min test), on average by 15.6 percentage points over the 3 sessions (Figure 3). This plot demonstrates that all subjects scored higher than the median score of a random note generator; only three tests from three different participants are slightly below the 95th percentile score of a random note generator (two subjects during Test 1 and one during Test 4).

A subjective self-reporting survey of ratings of such factors as enjoyment, feeling of expressing oneself, discomfort, and feeling of control showed a generally favorable response (Table 3). Subjects were asked at the end of the final session to rate subjective factors from 1 (completely disagree) to 5 (completely agree). Average ratings from 1 to 5 of the following factors were: enjoyment 4.5, expression 3.7, satisfaction 4.5, lack of physical discomfort 4.7, and lack of emotional discomfort 4.7; these show a generally favorable response. While responses were favorable, there was a favorable but slightly less positive level of agreement occurring with the statement of a feeling of expressing oneself (average of 3.70 out of 5). A general trend noted (subjective, not quantified) was subjects noticing a feeling of genuine control beginning at around 70% accuracy.

**Table 3:** Results of a survey of subject experience.

Subject	I enjoyed my experience with the Encephalophone	I felt that I was able to express myself somewhat with the Encephalophone	I felt that learning to use the Encephalophone was satisfying	The sessions did not cause physical discomfort to me	The sessions did not cause emotional discomfort to me
1	5	4	5	5	5
2	5	4	5	5	5
3	5	4	5	5	4
4	4	2	3	4	5
5	4	3	4	5	5
6	5	5	4	5	4
7	5	3	5	4	5
8	3	4	5	5	5
9	5	5	5	5	5
10	4	3	4	4	4
Average	4.50	3.70	4.50	4.70	4.70

Scale from 1 (completely disagree) to 5 (completely agree).

## Discussion

We demonstrate with this small pilot study that subjects with acquired motor disability in a hospital setting can operate the Encephalophone audio-based brain computer interface with accuracy significantly better than random. Subjects also were able to improve their control of the instrument - as measured by note-matching accuracy - through learning over 3 sessions. The 10 subjects on average improved accuracy percentage by 15.6 percentage points and increased number of hits by 58.7% over the 3 sessions, with all subjects scoring accuracy significantly above random probability (19.05%). Additionally, these subjects reported a favorable response in terms of feelings of enjoyment, lack of discomfort, and self-expression.

In our analysis, we have used simulations to estimate and provide uncertainty bounds for the level of accuracy which could be obtained on the Encephalophone by random chance, as well as verified that the study participants performed better than a random note generator would. One caveat of this comparison is that in the random note generator simulations we take independent draws from a uniform distribution. In reality, if an individual is connected to the Encephalophone and not trying to hit a specific target note, it is unlikely that the notes they emit would follow a completely independent uniform distribution. We also fit a mixed effects model for each of the two different measures of performance: number of hits and accuracy score. In both analyses, we found statistically significant evidence for improved performance over time. However, it appears that most of the increase in proficiency occurred in the last test. This result might suggest that there is an accumulated learning effect. Interestingly, previous studies (McCreadie et al., 2013; NIjboer et al., 2008) showed an initially better performance using visual feedback than auditory feedback, but an improvement and learning effect resulting in better performance with auditory feedback after several sessions.

It is significant that subjects with a wide range of etiologies for acquired motor disability (Table 1) all responded positively to the Encephalophone training, suggesting the potential versatility and broader utility of this novel tool, although the generalizability from a small pilot study is limited by the small sample size and heterogeneity of etiologies of disability. It would be interesting to broaden the range of disability or classification of neurologic injury to include aphasia and Chronic Traumatic Encephalopathy (CTE) in subjects who played sports where head contact was involved, or degenerative brain diseases such as Parkinson’s Disease, to identify limiting cases for efficacy of the approach. Studies of BCIs applied to patients with upper limb weakness from etiologies such as stroke have shown improvement in rehabilitation over traditional physical therapy alone (Khan et al., 2020; Wang et al., 2024), and a trial of the Encephalophone limited to subjects with upper-limb weakness from ischemic stroke may point to a specific and common etiology of acquired motor disability that may benefit. These more etiology-specific studies would allow us to refine the patient population for which BCI therapeutic approaches might have greatest value.

Future experiments with more sessions should demonstrate continued improvement in accuracy if the learning effect we observed is sustained. We hope to improve classification accuracy and reduce motor-imagery BCI ‘illiteracy’ (the approximate 15–20% of users who fail to achieve acceptable accuracy) through current development of algorithmic improvements of the Encephalophone using deep learning and brain-inspired neural networks (Wang et al., 2021; Arpaia et al., 2022; Hameed et al., 2025). Additionally with regards to visual feedback, the Encephalophone may be used as a basis for auditory-based BCIs for those with visual impairment, who might not be able to use a visual feedback-based BCI (Choi et al., 2023).

The Encephalophone has future promise for continued enablement of musical expression in those that have lost the ability to express themselves musically or otherwise (e.g., expressive language deficits). Most subjects express great satisfaction in being able to once again create music when they have lost this ability (as per questionnaire, Table 3), once they get a sense of genuine control. Nonetheless, this expressivity score of 3.7/5 on the questionnaire was slightly lower than other subjective scores (which are 4.5/5 to 4.7/5). Subjectively, we have observed that this sense of genuine control mostly begins at approximately 70% accuracy or higher, and this sense of control is likely very correlated with a sense of expressivity. The finding that these subjects can improve through learning over multiple sessions suggests that most subjects develop a sense of control of musical expression through the Encephalophone, given multiple sessions. However, three sessions may not be sufficient to get a comprehensive evaluation of a learning effect. Future longitudinal trials with more sessions may help determine if the learning effect is sustained and improvement continues (vs. a plateauing effect).

With this goal in mind, a portable version of the Encephalophone might be able to bring this expressive ability into the home of many more people (and for many more sessions) than is possible in the hospital in a clinical setting. Through the motivating power of music (Moens et al., 2014; Bergstrom et al., 2014; Maes et al., 2016), we hypothesize that more sustained learning sessions may be able to provide increasing accuracy and control to subjects over time than other modalities of feedback (e.g., visual feedback alone). With increasing accuracy, the Encephalophone may be able to add other control mechanisms (e.g., movement of a motorized prosthetic arm) to the musical feedback to enable subjects further: musical audio feedback is particularly motivating as feedback for movement (Van Dyck et al., 2015). Additionally, we hypothesize there may be significant cognitive benefits such as increased focus and executive function (Carelli et al., 2017; Angelakis et al., 2007), to provide therapeutic effect for those with cognitive impairments, Attention-Deficit/Hyperactivity Disorder (Lim et al., 2012) or Post-Traumatic Stress Disorder (van der Kolk et al., 2016) that the Encephalophone might provide. Individuals with speech and language might benefit in particular from increased expressive ability via the Encephalophone. These additional potential benefits should be further investigated in future studies with the Encephalophone.

## Data Availability

The raw data supporting the conclusions of this article will be made available by the authors, without undue reservation.

## References

[ref1] American Electroencephalographic Society (1994). Guideline thirteen: guidelines for standard electrode position nomenclature. J. Clin. Neurophysiol. 11, 111–113. 8195414

[ref2] Angelakis E. Stathopoulou S. Frymiare J. L. Green D. L. Lubar J. F. Kounios J. (2007). EEG neurofeedback: a brief overview and an example of peak alpha frequency training for cognitive enhancement in the elderly. Clin. Neuropsychol. 21, 110–129. doi: 10.1080/13854040600744839, PMID: 17366280

[ref3] Arpaia P. Esposito A. Natalizio A. Parvis M. (2022). How to successfully classify EEG in motor imagery BCI: a metrological analysis of the state of the art. J. Neural Eng. 19:031002. doi: 10.1088/1741-2552/ac74e0, PMID: 35640554

[ref4] Bergstrom I. Seinfeld S. Arroyo-Palacios J. Slater M. Sanchez-Vives M. V. (2014). Using music as a signal for biofeedback. Int. J. Psychophysiol. 93, 140–149. doi: 10.1016/j.ijpsycho.2013.04.013, PMID: 23623954

[ref5] Carelli L. Solca F. Faini A. Meriggi P. Sangalli D. Cipresso P.. (2017). Brain-computer interface for clinical purposes: cognitive assessment and rehabilitation. Biomed. Res. Int. 2017:1695290. doi: 10.1155/2017/1695290, PMID: 28913349 PMC5587953

[ref6] Choi Y. J. Kwon O. S. Kim S. P. (2023). Design of auditory P300-based brain-computer interfaces with a single auditory channel and no visual support. Cogn. Neurodyn. 17, 1401–1416. doi: 10.1007/s11571-022-09901-3, PMID: 37974580 PMC10640544

[ref7] Deuel T. A. Pampin J. Sundstrom J. Darvas F. (2017). The Encephalophone: a novel musical biofeedback device using conscious control of electroencephalogram (EEG). Front. Hum. Neurosci. 11:213. doi: 10.3389/fnhum.2017.00213, PMID: 28491030 PMC5405117

[ref8] Hameed I. Khan D. M. Ahmed S. M. Aftab S. S. Fazal H. (2025). Enhancing motor imagery EEG signal decoding through machine learning: a systematic review of recent progress. Comput. Biol. Med. 185:109534. doi: 10.1016/j.compbiomed.2024.109534, PMID: 39672015

[ref9] Khan M. A. Das R. Iversen H. K. Puthusserypady S. (2020). Review on motor imagery based BCI systems for upper limb post-stroke neurorehabilitation: from designing to application. Comput. Biol. Med. 123:103843. doi: 10.1016/j.compbiomed.2020.103843, PMID: 32768038

[ref10] Kiss L. Karina J. L. (2021). The effect of preferred background music on task-focus in sustained attention. Psychol. Res. 85, 2313–2325. doi: 10.1007/s00426-020-01400-6, PMID: 32748062 PMC8357712

[ref11] Lim C. G. Lee T. S. Guan C. Fung D. S. Zhao Y. Teng S. S.. (2012). A brain-computer interface based attention training program for treating attention deficit hyperactivity disorder. PLoS One 7:e46692. doi: 10.1371/journal.pone.0046692, PMID: 23115630 PMC3480363

[ref12] Maes P. J. Buhmann J. Leman M. (2016). 3Mo: a model for music-based biofeedback. Front. Neurosci. 10:548. doi: 10.3389/fnins.2016.00548, PMID: 27994535 PMC5133250

[ref13] McCreadie K. A. Coyle D. H. Prasad G. (2013). Sensorimotor learning with stereo auditory feedback for a brain–computer interface. Med. Biol. Eng. Comput. 51, 285–293. doi: 10.1007/s11517-012-0992-7 23197181

[ref14] Moens B. Muller C. van Noorden L. Franěk M. Celie B. Boone J.. (2014). Encouraging spontaneous synchronisation with D-jogger, an adaptive music player that aligns movement and music. PLoS One 9:e114234. doi: 10.1371/journal.pone.0114234, PMID: 25489742 PMC4260851

[ref15] Neuper C. Müller-Putz G. R. Scherer R. Pfurtscheller G. (2006). Motor imagery and EEG-based control of spelling devices and neuroprostheses. Prog. Brain Res. 159, 393–409. doi: 10.1016/S0079-6123(06)59025-9, PMID: 17071244

[ref16] Nijboer F. Birbaume N. Kübler A. (2010). The influence of psychological state and motivation on brain–computer interface performance in patients with amyotrophic lateral sclerosis – a longitudinal study. Front. Neurosci. 4:55. doi: 10.3389/fnins.2010.00055 20700521 PMC2916671

[ref17] Nijboer F. Furdea A. Gunst I. Mellinger J. McFarland D. J. Birbaumer N.. (2008). An auditory brain-computer interface (BCI). J. Neurosci. Methods 167, 43–50. doi: 10.1016/j.jneumeth.2007.02.009 17399797 PMC7955811

[ref18] van der Kolk B. A. Hodgdon H. Gapen M. Musicaro R. Suvak M. K. Hamlin E.. (2016). A randomized controlled study of neurofeedback for chronic PTSD. PLoS One 11:e0166752. doi: 10.1371/journal.pone.0166752, PMID: 27992435 PMC5161315

[ref19] Van Dyck E. Moens B. Buhmann J. Demey M. Coorevits E. Dalla Bella S.. (2015). Spontaneous entrainment of running cadence to music tempo. Sports Med. Open 1:15. doi: 10.1186/s40798-015-0025-9, PMID: 26258007 PMC4526248

[ref20] Wang T. Du S. Dong E. (2021). A novel method to reduce the motor imagery BCI illiteracy. Med. Biol. Eng. Comput. 59, 2205–2217. doi: 10.1007/s11517-021-02449-0, PMID: 34674118

[ref21] Wang A. Tian X. Jiang D. Yang C. Xu Q. Zhang Y.. (2024). Rehabilitation with brain-computer interface and upper limb motor function in ischemic stroke: a randomized controlled trial. Med 5, 559–569.e4. doi: 10.1016/j.medj.2024.02.014, PMID: 38642555

